# Supplementary material for: Temporal Dynamics of High-Density Lipoprotein Proteome in Diet-Controlled Subjects with Type 2 Diabetes
Source: Biomolecules. 2020 Mar 30;10(4):520. doi: 10.3390/biom10040520 (PMC7226298; doi:10.3390/biom10040520)
Supplement: Supplementary file 1 [file biomolecules-10-00520-s001.pdf]

Online Supplemental Materials

Detailed Methods

Materials.
High-purity ^2H_2O was obtained from the Cambridge Isotope Laboratory (Andover, MA). All other chemicals were from Sigma-Aldrich. Before administration, the ^2H_2O was boiled and filtered through a sterile filter.

Subjects.

All healthy individuals were recruited by advertisement and/or targeted search within the electronic medical records at Cleveland Clinic, Ohio. Prior to HDL flux studies in patients with T2DM and age- and BMI-matched healthy controls, we studied HDL metabolism in healthy young adults to optimize the ^2H_2O-metabolic labeling protocol. Each potential subject underwent medical screening that included a physical examination and blood chemistry profile. Patients with T2DM were recruited at the endocrinology department of Cleveland Clinic. All patients were newly-diagnosed insulin-naive individuals and were not taking any oral hypoglycemic drugs with an average diabetes duration of 1-3 months. These patients were diagnosed based on oral glucose tolerance test (≥ 200 mg/dL after 2 h of 75 g dextrose challenge) and HbA_{1c} (> 6.5) or both as defined by the American Diabetes Association's criteria. Since diagnosis, all patients with T2DM were advised to adhere to lifestyle modification (hypocaloric, carbohydrate-controlled diet and moderate physical activity). Follow-up counseling was provided by endocrinologists. Individuals were excluded if they had undergone significant weight loss (> 2 kg), or had been engaged in intensive physical activity in the previous 6 months, had history of alcohol and/or drug abuse, were smokers or had quit smoking within past 3 months, or if they showed any evidence of cardiovascular, renal, hepatic, hypothyroid or hematological diseases. In addition, we excluded all subjects on any lipid-lowering drugs, β-blockers or agents known to affect lipid metabolism. Following medical screening, 8 adult (4 women and 4 men, age 50.7 ± 11.6 years), overweight (BMI 28.7 ± 3.1 kg/m^2) healthy control subjects and 9 age- and BMI-matched patients with diet-controlled T2DM (5 women and 4 men) were enrolled to the HDL turnover study. The Cleveland Clinic's Institutional Review Board reviewed and approved the protocols. All volunteers gave their informed written consent to partake in the study after having the procedures and potential risks fully explained. For the three days immediately prior to, and during the one week HDL turnover study, all subjects were advised to avoid strenuous exercise and to consume an isocaloric diet to ensure weight stability and prevent any diet- and exercise-induced changes in HDL metabolism. Each subject underwent an HDL turnover study, as described below.

Analytical Procedures and enzyme activity assays

HDL turnover: HDL turnover was assessed in ApoB-depleted serum using the ^2H_2O-metabolic labeling approach as described [1]. Briefly, serum (30 µL) was diluted with one volume of PBS and centrifuged at 21300 g for 3 hours at 4 ºC to spin up VLDL. The lower phase (20 µL) of the emulsion was removed and ApoB-containing particles (IDL and LDL) were precipitated with 3 µL of a magnesium chloride/dextran sulfate reagent (Stanbio Laboratory, Boerne, TX) [2]. The sample was then centrifuged at 14000 g for 10 minutes at 4 ºC. The supernatant containing ApoB-depleted serum was recovered and used for the analysis of both HDLc and ApoAI. HDL proteins, including ApoAI, were precipitated with 1 mL of cold acetone at -20 ºC for 4 hours and then centrifuged at 2000 g for 5 minutes. The pellet was saved for the analysis of ApoAI and other proteins. The supernatant was used for HDLc analysis. For this purpose, the solvent was evaporated and the dried residue was treated with 1M potassium hydroxide in 70% ethanol to hydrolyze cholesteryl esters. Total cholesterol was extracted with pentane and after evaporation of solvent, the residue was derivatized with the trimethylchlorosilane (TMS) reagent. The ^{2}H-enrichment of cholesterol was determined using an Agilent

5977A-MSD mass spectrometer equipped with an Agilent 6890B GC system. Electron impact ionization (70 eV) with selected ion monitoring (SIM) of m/z 368-371 (M_0-M_3 cholesterol) dwell time of 10 millisecond per ion was used for all analyses [1]. The time course ^{2}H enrichment of cholesterol was used for the kinetic analysis as described below.

The pellet isolated from ApoB-depleted plasma was washed three times with cold acetone and centrifuged. Proteins were denaturated by the addition of 100 µL of 6 M urea solution in 100 mM Tris buffer (pH 8) prepared in deionized water at 4 ºC overnight. To reduce the disulfide bonds of the proteins, the samples were reacted with dithiothreitol (DTT) (9 µL, 30 mg/ml in 100 mM pH 8 Tris buffer) for 20 minutes at room temperature and then free thiols were alkylated with an excess of 2-iodoacetamide (9 µL of 36 mg/ml solution in 100 mM pH 8 Tris buffer) for 20 minutes at room temperature. Proteins were digested in solution with an excess of Promega sequencing grade trypsin (10 µL of 100 ng/µL trypsin solution in 100 mM pH 8 Tris buffer) at room temperature overnight. Sample was desalted using a Pierce C18 Pepclean solid-phase extraction spin-column. Peptides were eluted with 2 x 20 µL 70% acetonitrile and the solvent was evaporated under vacuum. Samples were reconstituted in 30 µL of 0.1% formic acid and 5 µL of this solution was injected for LC-MS/MS analysis [3].

Proteomics analyses: Chromatographic separation of the protein digest was performed on an UltiMate-3000 Rapid Separation LC instrument (Thermo Fisher Scientific, Bremen, Germany). Tryptic peptides were loaded onto a PepMap trapping column (C18, 100 µm×5 mm, Thermo Fisher). Samples were desalted for 5 minutes with water and 0.1% TFA at the flow rate of 10 µl/min. Reverse-phase separation of peptides was then performed on an analytical C18 PepMap column (75 μm × 15 cm, Thermo Fisher) using mobile phases A (0.1% formic acid in water) and B (80% acetonitrile and 0.1% formic acid in water) with a linear gradient starting at 5% B and then up to 35% at 150 min at a flow rate of 300 nl/min. Mobile phase B then ramped to 80% in 5 min and then held at 80% for 15 min. Eluted peptides were ionized via a non-coated nano-spray emitter (10 µm, FS360-20-10-N-5-105CT, New Objective Inc., Woburn, MA) in positive mode at a voltage of 2.2 kV. Inlet capillary temperature was maintained at 250 ºC.

Mass spectrometry analysis was performed on a Q Exactive Plus (Thermo Fisher Scientific) instrument using Xcalibur 2.2 software. Each full MS scan was followed by the top 10 high-energy collisional dissociation (HCD) MS/MS scans. Full scans (m/z 380-1300) were acquired at a resolution of 70,000 (at m/z 200) and a targeted automatic gain control (AGC) value of 1 x 10^6. MS/MS scans were performed at a resolution of 17500 (m/z 200) and AGC target of 2 x 10^4 ions. Precursor ions were isolated with an isolation window of 1.4 m/z and fragmented at 27 eV. Product ion spectra were acquired at a starting mass of m/z 140. Maximum fill times were 100 ms for MS and MS/MS scans and dynamic exclusion was enabled for a duration of 17 seconds.

To improve the sensitivity and stability of less abundant signals, ions of interest were recorded both in full scan and in selected-ion monitoring (SIM) modes, in parallel. The SIM scan monitored 75 selected ions including a list of native and glycated peptides with an individual isolation window of 10 Da at a resolution of 70,000, a target AGC value of 1 x 10^6, and a maximum individual fill time of 200 ms.

Isotope incorporation was assessed based on mass isotopomer distribution analysis of the high-resolution full-scan spectra as described previously [4]. Mass isotopomers are molecules that differ by the presence of different heavy isotopes resulting in a mass spectrum with a baseline monoisotopic (M_0) peak followed by distinct heavy isotopomer (M_i, where i is an integer > 0) peaks. Only high-abundance ions (10^5-10^7 intensity) were selected for accurate quantification of isotope incorporation. Peaks that exhibited a Gaussian distribution and had no interference with isobaric peaks were used for the analysis. Quantification was performed by integrating each isotopomer of a given chromatographic peak within a defined mass range (20 ppm). The kinetics of a protein was analyzed using the isotopic distribution of its tryptic unique peptides.

Supplementary Table 1. List of the identified proteins in HDL isolated by anti-HDL immunoaffinity method in healthy controls and T2D patients (n=8/group).

Protein #	Accession Number	Protein Name	Newly identified proteins
1	P02787	Serotransferrin	
2	P27169	Serum paraoxonase/arylesterase 1	
3	P02647	Apolipoprotein A-I	
4	O14791	Apolipoprotein L1	
5	O95445	Apolipoprotein M	
6	P02649	Apolipoprotein E	
7	P02652	Apolipoprotein A-II	
8	P02655	Apolipoprotein C-II	
9	P02656	Apolipoprotein C-III	
10	P04114	Apolipoprotein B-100	
11	P08519	Apolipoprotein(a)	
12	P02654	Apolipoprotein C-I	
13	Q13790	Apolipoprotein F	
14	P00736	Complement C1r subcomponent	
15	P01024	Complement C3	
16	P02746	Complement C1q subcomponent subunit B	x
17	P02748	Complement component C9	
18	P07358	Complement component C8 beta chain	
19	P07360	Complement component C8 gamma chain	x
20	P08603	Complement factor H	
21	P09871	Complement C1s subcomponent	
22	P0C0L4	Complement C4-A	
23	P0C0L5	Complement C4-B	
24	P00738	Haptoglobin	
25	P00739	Haptoglobin-related protein	
26	P02766	Transthyretin	
27	P02790	Hemopexin	
28	O75636	Ficolin-3	x
29	P00488	Coagulation factor XIII A chain	x
30	P00734	Prothrombin	
31	P00747	Plasminogen	
32	P00748	Coagulation factor XII	x
33	P01008	Antithrombin-III	
34	P01009	Alpha-1-antitrypsin	
35	P01011	Alpha-1-antichymotrypsin	
36	P01023	Alpha-2-macroglobulin	

37	P01042	Kininogen-1
38	P01834	Ig kappa chain C region
39	P01857	Ig gamma-1 chain C region
40	P01871	Ig mu chain C region
41	P01876	Ig alpha-1 chain C region
42	P02749	Beta-2-glycoprotein 1
43	P02751	Fibronectin
44	P02760	Protein AMBP
45	P02765	Alpha-2-HS-glycoprotein
46	P02768-1	Serum albumin
47	P02776	Platelet factor 4
48	P03952	Plasma kallikrein
49	P04003	C4b-binding protein alpha chain
50	P04004	Vitronectin
51	P04070	Vitamin K-dependent protein C
52	P04196	Histidine-rich glycoprotein
53	P04275	von Willebrand factor
54	P04406	Glyceraldehyde-3-phosphate dehydrogenase
55	P05090	Apolipoprotein D
56	P05546	Heparin cofactor 2
57	P06727	Apolipoprotein A-IV
58	P07225	Vitamin K-dependent protein S
59	P08697	Alpha-2-antiplasmin
60	P10909	Clusterin
61	P18428	Lipopolysaccharide-binding protein
62	P19652	Alpha-1-acid glycoprotein 2
63	P19823	Inter-alpha-trypsin inhibitor heavy chain H2
64	P19827	Inter-alpha-trypsin inhibitor heavy chain H1
65	P20742	Pregnancy zone protein
66	P22792	Carboxypeptidase N subunit 2
67	P23142	Fibulin-1
68	P35542	Serum amyloid A-4 protein
69	P55058	Phospholipid transfer protein
70	P80108	Phosphatidylinositol-glycan-specific phospholipase D
71	Q08380	Galectin-3-binding protein
72	Q14624	Inter-alpha-trypsin inhibitor heavy chain H4
73	Q15166	Serum paraoxonase/lactonase 3
74	P29622	Kallistatin
75	Q6P0F9	Insulinoma-associated protein 1a
76	Q8IID4	Dynein heavy chain-like protein PF11_0240
77	Q8IZ40	REST corepressor 2

78	Q96IY4	Carboxypeptidase B2
79	Q96KN2	Beta-Ala-His dipeptidase
80	Q96PD5	N-acetylmuramoyl-L-alanine amidase
81	Q9GLN8	Angiotensinogen
82	Q9HDC9	Adipocyte plasma membrane-associated protein
83	Q9UHG3	Prenylcysteine oxidase 1

Supplementary Table 2. Comparison of the half-lives of HDL proteins in healthy controls and T2D patients isolated by anti-HDL immunocapture and ApoB-depletion methods. To assess whether each method could detect the effect of diabetes on HDL proteome dynamics, we processed samples from a T2D patient and an age- and BMI-matched healthy controls. Consistent with the removal of ApoE[5] by the dextran sulfate/$MgCl_2$ approach, ApoE kinetics was quantified using only the immunocapture method. In addition, the immunocapture method also enabled isolation and quantification of the kinetics of SAA IV, a key protein involved in pro-inflammatory remodeling of HDL. Data present mean ± SD. Mean values of half-life for each protein were calculated as the average of the half-lives of multiple unique peptides corresponding to that protein. * Half-life is calculated based on only one peptide.

Accession Number	Protein Name	CONTROL		T2DM	
		Immunocapture t ½ (hr)	ApoB-depletion t ½ (hr)	Immunocapture t ½ (hr)	ApoB-depletion t ½ (hr)
**P01024**	Complement C3	61.9±5.8	62.4±16.3	42.0±10.6	43.5±6.5
**P10909**	Clusterin	24.0±2.3	18.2±9.8	17.0±1.7	19.2±4.8
**P27169**	Paraoxonase/ arylesterase 1	212.0±33.5	208.1*	170.1±28.5	187.0*
**P02647**	Apolipoprotein AI	110.3±7.6	107.6±15.1	42.4±7.0	43.5±5.0
**P02652**	Apolipoprotein AII	131.8±17.8	130.1±30.5	67.8±7.9	67.9±23.5
**P06727**	Apolipoprotein AIV	49.3±11.7	49.0±10.7	41.9±14.8	40.6±5.3
**P02656**	Apolipoprotein CIII	21.6±1.1	22.3±3.1	20.8±1.7	22.4±3.4
**P02649**	Apolipoprotein E	15.7±2.8	N/A	22.7±6.9	N/A
	Serum amyloid amylase IV	112.3±8.92	N/A	28.1±0.7	N/A
**P00734**	Prothrombin	49.5±6.8	47.9±6.3	49.4±8.4	50.1±10.0
**P02766**	Transheretin	32.0±5.4	32.2±4.9	24.3±3.7	27.8±4.3
**P00739**	Haptoglobin-related protein	92.2±16.3	95.6±13.2	39.1±12.2	42.5±5.0

References:

1. Kasumov T, Willard B, Li L, Li M, Conger H, Buffa JA, Previs S, McCullough A, Hazen SL and Smith JD. (H2O)-H-2-Based High-Density Lipoprotein Turnover Method for the Assessment of Dynamic High-Density Lipoprotein Function in Mice. *Arterioscl Throm Vas*. 2013;33:1994-2003.
2. Finley PR, Schifman RB, Williams RJ and Lichti DA. Cholesterol in high-density lipoprotein: use of Mg2+/dextran sulfate in its enzymic measurement. *Clinical chemistry*. 1978;24:931-3.
3. Li L, Willard B, Rachdaoui N, Kirwan JP, Sadygov RG, Stanley WC, Previs S, McCullough AJ and Kasumov T. Plasma Proteome Dynamics: Analysis of Lipoproteins and Acute Phase Response Proteins with (H2O)-H-2 Metabolic Labeling. *Mol Cell Proteomics*. 2012;11.
4. Kasumov T, Dabkowski ER, Shekar KC, Li L, Ribeiro RF, Walsh K, Previs SF, Sadygov RG, Willard B and Stanley WC. Assessment of cardiac proteome dynamics with heavy water: slower protein synthesis rates in interfibrillar than subsarcolemmal mitochondria. *Am J Physiol-Heart C*. 2013;304:H1201-H1214.
5. Davidson WS, Heink A, Sexmith H, Melchior JT, Gordon SM, Kuklenyik Z, Woollett L, Barr JR, Jones JI, Toth CA and Shah AS. The effects of apolipoprotein B depletion on HDL subspecies composition and function. *J Lipid Res*. 2016;57:674-686.
